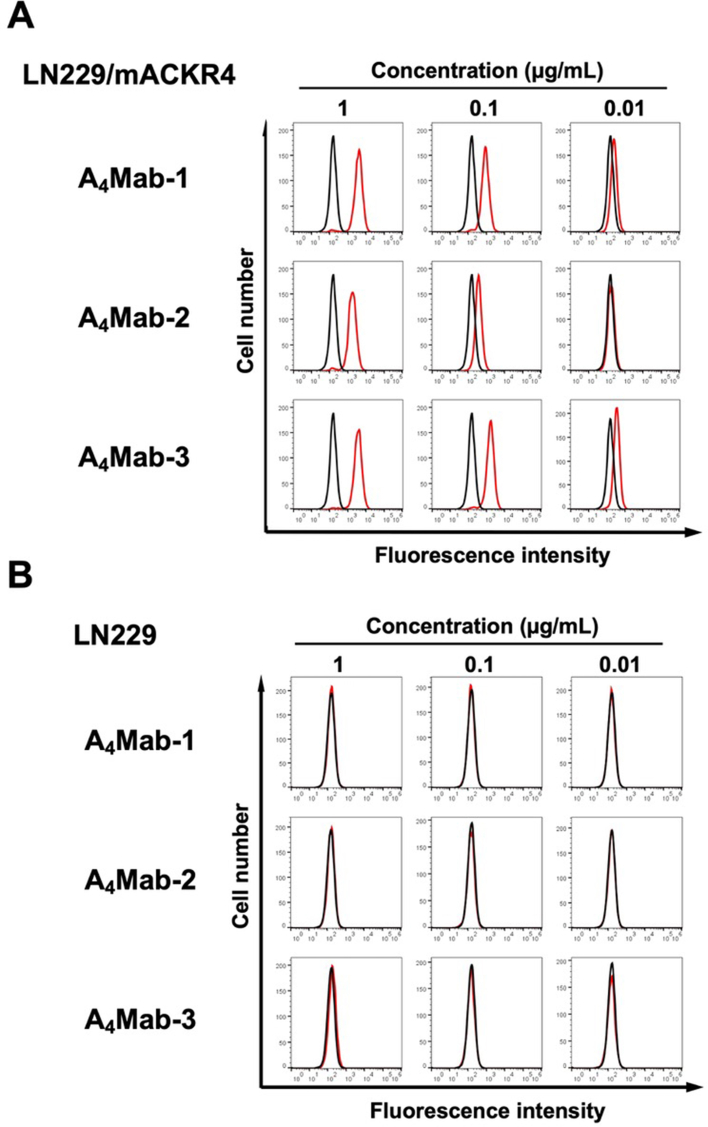# Development of specific anti-mouse atypical chemokine receptor 4 monoclonal antibodies

**DOI:** 10.1016/j.bbrep.2024.101824

**Published:** 2024-09-07

**Authors:** Miu Hirose, Hiroyuki Suzuki, Rena Ubukata, Tomohiro Tanaka, Mika K. Kaneko, Yukinari Kato

**Affiliations:** Department of Antibody Drug Development, Tohoku University Graduate School of Medicine, 2-1 Seiryo-machi, Aoba-ku, Sendai, 980-8575, Miyagi, Japan

**Keywords:** Mouse ACKR4, Monoclonal antibody, Peptide immunization, Flow cytometry, Western blotting

## Abstract

Leukocyte migration is an essential function of innate and adaptive immune responses. Chemokines and their receptors control the migration system. The abundance of chemokines is controlled by atypical chemokine receptors (ACKRs), chemokine receptor-like molecules that do not couple to the G protein signaling pathways. Among them, ACKR4 regulates dendritic cell migration by controlling the ligands and is involved in tumor development in mouse models. Because no anti-mouse ACKR4 (mACKR4) monoclonal antibody (mAb) for flow cytometry has been reported, this study aimed to develop a novel mAb for mACKR4. Among the established anti-mACKR4 mAbs, A_4_Mab-1 (rat IgG_2b_, kappa), A_4_Mab-2 (rat IgG_2b_, kappa), and A_4_Mab-3 (rat IgG_2b_, kappa) recognized mACKR4-overexpressed Chinese hamster ovary-K1 (CHO/mACKR4) by flow cytometry. The dissociation constant (*K*_D_) values of A_4_Mab-1, A_4_Mab-2, and A_4_Mab-3 for CHO/mACKR4 were determined as 6.0 × 10^−9^ M, 1.3 × 10^−8^ M, and 1.7 × 10^−9^ M, respectively. Furthermore, A_4_Mab-1 and A_4_Mab-2 could detect mACKR4 by western blotting. These results indicated that A_4_Mab-1, A_4_Mab-2, and A_4_Mab-3 help to detect mACKR4 by flow cytometry and western blotting and obtain the proof of concept in preclinical models.

## Highlights

• Novel anti-mACKR4 mAbs were developed by the N-terminal peptide immunization.
• Three anti-mACKR4 mAbs are useful for flow cytometry or western blotting.
• Anti-mACKR4 mAbs could contribute to the preclinical studies for tumor immunotherapy.

## 1 Introduction

The immune cell priming, memory responses, and effector functions are controlled by chemokines and the restricted expression of the G protein-coupled receptors (GPCRs) [1]. The chemokine receptors are the most prominent family of receptors, which possess a seven-transmembrane domain. They can be categorized into a larger subgroup of G protein-coupled receptors and a smaller subgroup of atypical chemokine receptors (ACKR1 to ACKR4) [2,3]. Additional candidates designated as CCRL2 (ACKR5), PITPNM3 (ACKR6), GPR182, and CXCR3-B are identified, but they are required for further functional characterization [2].

Upon a chemokine binding to its GPCR, the G protein subunits are usually activated [4]. The ACKRs are homologous to GPCRs. However, ACKRs fail in inducing the classical signaling through G protein [2]. Instead, the signaling is transduced to β-arrestin-dependent internalization of receptor and ligand, which leads to degradation of chemokine [2]. Therefore, ACKRs function as a chemokine scavenger receptor that mediates chemokines' rapid internalization and degradation.

ACKR4 has been described as expressed in not only T lymphocytes [5], but also stromal cells [6,7]. ACKR4 binds to the chemokines, including CCL19, CCL20, CCL21, CCL22, and CCL25. ACKR4 regulates migratory responses driven through CCR7 (activated by CCL19 and CCL21), CCR6 (activated by CCL20), CCR4 (activated by CCL22), and CCR9 (activated by CCL25) [2]. Therefore, ACKR4 controls the bioavailability of the abovementioned chemokines by creating a chemokine gradient, which facilitates the directional migration of dendritic cells (DCs) from the non-lymphatic tissue to the draining lymph node [6,8,9]. ACKR4 is also expressed in a flow-dependent manner in afferent lymphatic collectors. The ACKR4 removes CCL21 from the collector surface which prevents T cell migration to inflamed dermal collectors. In the absence of ACKR4, the T cell migration to draining lymph nodes is reduced [10].

ACKR4 expression is reduced in human colorectal cancer (CRC) compared with normal colon epithelial cells [11]. The downregulation of ACKR4 in CRC is associated with a weak antitumor immune response [11]. Loss of ACKR4 in mouse colorectal cancer cells impairs the DC migration to the tumor-draining lymph nodes, which leads to the reduced number of tumor-specific T-cells and resistance to immune checkpoint blockades [11]. In the MMTV-PyMT transgenic mouse model, which recapitulates the step-wise progression of human breast cancer [12], the loss of mACKR4 led to an increase in intratumor CD8^+^ T cells and CD103^+^ DCs and inhibited tumor development [12]. These findings promise further study of the targeting of ACKR4 to improve the efficacy of immunotherapy. Therefore, a specific monoclonal antibody (mAb) against mouse ACKR4 (mACKR4) is essential to identify and target the ACKR4-expressing cells in the preclinical tumor models.

We have employed the Cell-Based Immunization and Screening (CBIS) method and developed anti-mouse chemokine receptor mAbs against CCR1 (clone C_1_Mab-6) [13], CCR3 (clones C_3_Mab-2, C_3_Mab-3, and C_3_Mab-4) [[14], [15], [16]], CCR5 (clone C_5_Mab-2) [17], CCR8 (clones C_8_Mab-1, C_8_Mab-2, and C_8_Mab-3) [[18], [19], [20]], CXCR1 (clone Cx_1_Mab-1) [21], CXCR3 (clone Cx_3_Mab-4) [22], and CXCR4 (clone Cx_4_Mab-1) [23]. Furthermore, we established anti-mouse chemokine receptor mAbs against CCR2 (clone C_2_Mab-6) [24], CCR3 (clones C_3_Mab-6 and C_3_Mab-7) [25], CCR4 (clone C_4_Mab-1) [26], CCR5 (clones C_5_Mab-4 and C_5_Mab-8) [27], CCR9 (clone C_9_Mab-24) [28], and CXCR6 (clone Cx_6_Mab-1) [29] using the N-terminal peptide immunization. Because no anti-mACKR4 mAb was commercially available, we aimed to develop novel anti-mACKR4 mAbs.

## 2 Materials and methods

### 2.1 Cell lines and plasmids

P3X63Ag8U.1 (P3U1), LN229, and Chinese hamster ovary (CHO)–K1 cell lines were obtained from the American Type Culture Collection (Manassas, VA).

The synthesized mACKR4 (Accession No.: NM_145700.2) cDNA (Eurofins Genomics KK, Tokyo, Japan) was subsequently subcloned into pCAGzeo-nMAP vectors (FUJIFILM Wako Pure Chemical Corporation, Osaka, Japan) and was deposited to RIKEN Bioresource Center (Tsukuba, Japan). The MAP tag can be detected by an anti-mouse podoplanin mAb (clone PMab-1) [30]. The mACKR4 plasmids were transfected into CHO–K1 and LN229. Stable clones were sorted using a cell sorter (SH800; Sony Corp., Tokyo, Japan). These cells were cultured as described previously [29].

### 2.2 Peptides

The N-terminal extracellular region of mACKR4 (_1_-MALELNQSAEYYYEENEMN-_19_) plus C-terminal cysteine was synthesized by Eurofins Genomics KK. The keyhole limpet hemocyanin (KLH) was subsequently conjugated at the C-terminus.

### 2.3 Production of hybridomas

The approval of animal experiments was obtained from the Animal Care and Use Committee of Tohoku University (Permit number: 2022MdA-001). Two five-week-old Sprague-Dawley rats (CLEA Japan, Tokyo, Japan) were intraperitoneally immunized with 100 μg of the KLH-conjugated mACKR4 peptide (mACKR4-KLH) with Alhydrogel adjuvant 2 % (InvivoGen). After three additional weekly immunizations (100 μg/rat) and final booster immunizations (100 μg/rat), hybridomas were produced and cultured in the medium containing hypoxanthine, aminopterin, and thymidine (HAT; Thermo Fisher Scientific Inc., Waltham, MA), 10 % FBS, and 5 % BriClone (NICB, Dublin, Ireland). The supernatants were screened using enzyme-linked immunosorbent assay (ELISA) using the mACKR4 peptide. The supernatants were further screened by flow cytometry using CHO–K1 and CHO/mACKR4. Established anti-mACKR4 mAbs can be obtained from the Antibody Bank of Tohoku University (http://www.med-tohoku-antibody.com/topics/001_paper_antibody_PDIS.htm#ACKR4).

### 2.4 Purification of antibodies

The cultured supernatants of A_4_Mab-1, A_4_Mab-2, and A_4_Mab-3 hybridomas were purified using 1 mL of Ab-Capcher (ProteNova, Kagawa, Japan). The mAbs were eluted with an IgG elution buffer (Thermo Fisher Scientific Inc.), and replaced with phosphate-buffered saline (PBS) using Amicon Ultra (Merck KGaA, Darmstadt, Germany).

### 2.5 ELISA

The synthesized peptide (MALELNQSAEYYYEENEMNC) was immobilized on 96 well immunoplates (Thermo Fisher Scientific Inc.). Blocking was performed with 1 % bovine serum albumin (BSA)-PBS containing 0.05 % Tween20 (PBST; Nacalai Tesque, Inc.). The plates were incubated with supernatants, followed by peroxidase-conjugated anti-rat IgG (Sigma-Aldrich Corp., St. Louis, MO). The peroxidase reactions were performed using ELISA POD Substrate TMB Kit (Nacalai Tesque, Inc.).

### 2.6 Flow cytometric analysis

Cells were harvested after brief exposure to 1 mM EDTA. The cells were washed with blocking buffer (0.1 % BSA in PBS) and treated with 1, 0.1, and 0.01 μg/mL of A_4_Mab-1, A_4_Mab-2, and A_4_Mab-3 for 30 min at 4 °C. For peptide inhibition assay, A_4_Mab-1, A_4_Mab-2, and A_4_Mab-3 (0.1 μg/mL) were pre-incubated with 10 μg/mL of mACKR4 peptide or dimethyl sulfoxide (DMSO) for 25 min at 4 °C, and incubated with the cells for 30 min at 4 °C. Alexa Fluor 488-conjugated anti-rat IgG was used as a secondary antibody. The SA3800 Cell Analyzer (Sony Corp.) was used for fluorescence data collection and analysis. The dissociation constant (*K*_D_) was determined as described previously [27].

### 2.7 Western blotting

Sodium dodecyl sulfate-treated cell lysates from LN229 and mACKR4-overexpressed LN229 (LN229/mACKR4) were separated on polyacrylamide gels. The proteins were transferred onto polyvinylidene difluoride membranes (Merck KGaA). The membranes were incubated with 1 μg/mL of A_4_Mab-1, A_4_Mab-2, A_4_Mab-3, or AC-15 (an anti-β-actin mAb; Sigma-Aldrich Corp.). For peptide blocking assay, 1 μg/mL of A_4_Mab-1 and A_4_Mab-2 were pre-incubated with 1 μg/mL of mACKR4 peptide or DMSO for 15 min and incubated with the membranes. Peroxidase-conjugated anti-rat IgG or anti-mouse IgG (Agilent Technologies Inc., Santa Clara, CA) were used as secondary antibodies. The signals were detected using ImmunoStar LD (FUJIFILM Wako Pure Chemical Corporation) and a Sayaca-Imager (DRC Co. Ltd., Tokyo, Japan).

## 3 Results

### 3.1 Development of anti-mACKR4 mAbs using N-terminal peptide immunization

To develop anti-mACKR4 mAbs, two rats were immunized with mACKR4-KLH (Supplementary Fig. 1A). The splenocytes were fused with P3U1 myeloma cells and seeded into 96-well plates. Then, positive wells for the naked mACKR4 peptide were selected using ELISA. Then, CHO/mACKR4-reactive and CHO–K1-non-reactive supernatants were selected using flow cytometry (Supplementary Fig. 1B). After the cloning by limiting dilution from the independent wells and additional screenings, anti-mACKR4 mAbs, A_4_Mab-1 (rat IgG_2b_, kappa), A_4_Mab-2 (rat IgG_2b_, kappa), and A_4_Mab-3 (rat IgG_2b_, kappa) were finally established (Supplementary Fig. 1C). A_4_Mab-1 was first established from a series of immunization and screening. A_4_Mab-2 and A_4_Mab-3 were obtained from another one.

### 3.2 Flow cytometric analysis using A_4_Mab-1, A_4_Mab-2, and A_4_Mab-3

**Fig. 1:**
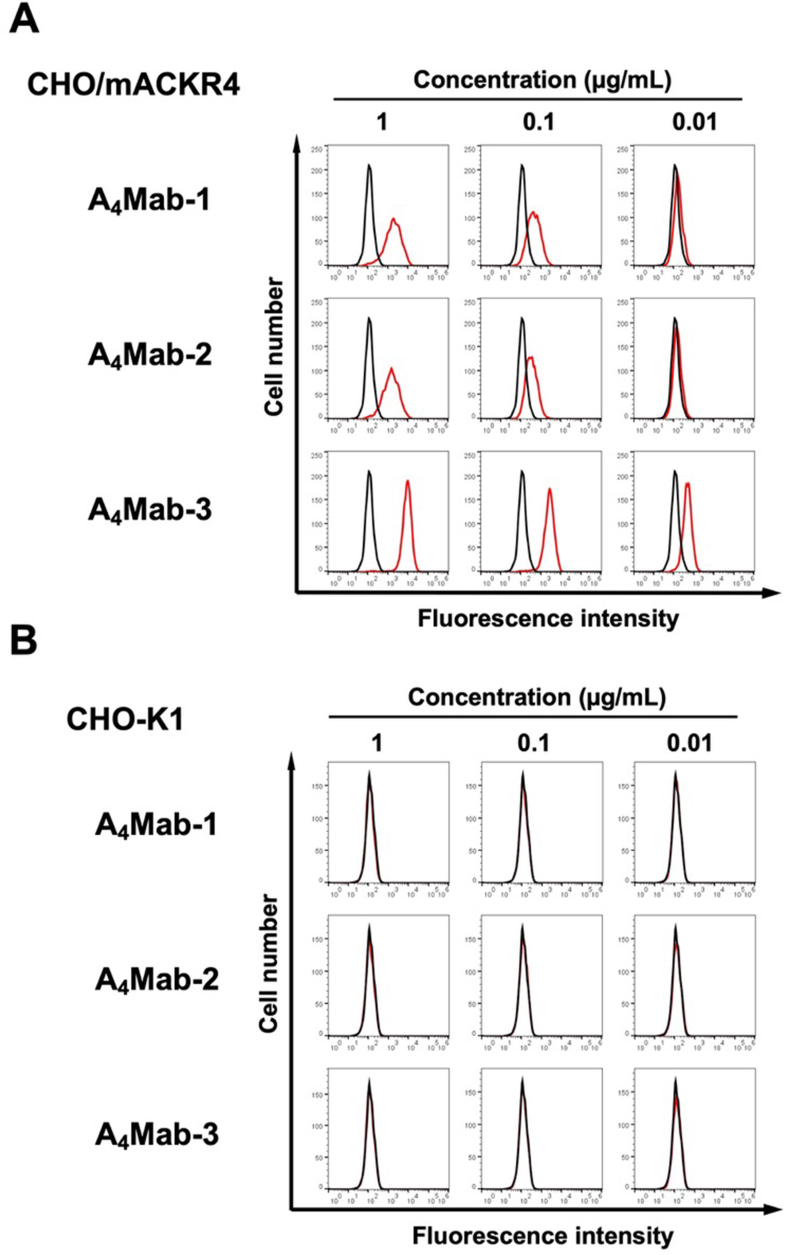
**Flow cytometry of mACKR4-overexpressed CHO–K1 cells using anti-mACKR4 mAbs.** CHO/mACKR4 (**A**) and CHO–K1 (**B**) cells were treated with 0.01–1 μg/mL of A_4_Mab-1, A_4_Mab-2, or A_4_Mab-3 (red line). The mAbs-treated cells were further incubated with anti-rat IgG conjugated with Alexa Fluor 488. The black line represents the negative control (blocking buffer). The dose-dependent reactivities of A_4_Mabs to CHO/mACKR4 were investigated at least three times.

We conducted flow cytometry using three anti-mACKR4 mAbs: A_4_Mab-1, A_4_Mab-2, and A_4_Mab-3 against CHO/mACKR4 and CHO–K1 cells. A_4_Mab-1, A_4_Mab-2, and A_4_Mab-3 recognized CHO/mACKR4 cells dose-dependently at 1, 0.1, and 0.01 μg/mL (Fig. 1A). A_4_Mab-3 exhibited a superior reactivity against CHO/mACKR4 cells compared to A_4_Mab-1 and A_4_Mab-2 (Fig. 1A). Parental CHO–K1 cells were not recognized by any mAbs even at 1 μg/mL (Fig. 1B). The superior reactivity of A_4_Mab-3 was also observed in LN229/mACKR4 cells (Supplementary Fig. 2).

**Fig. 2:**
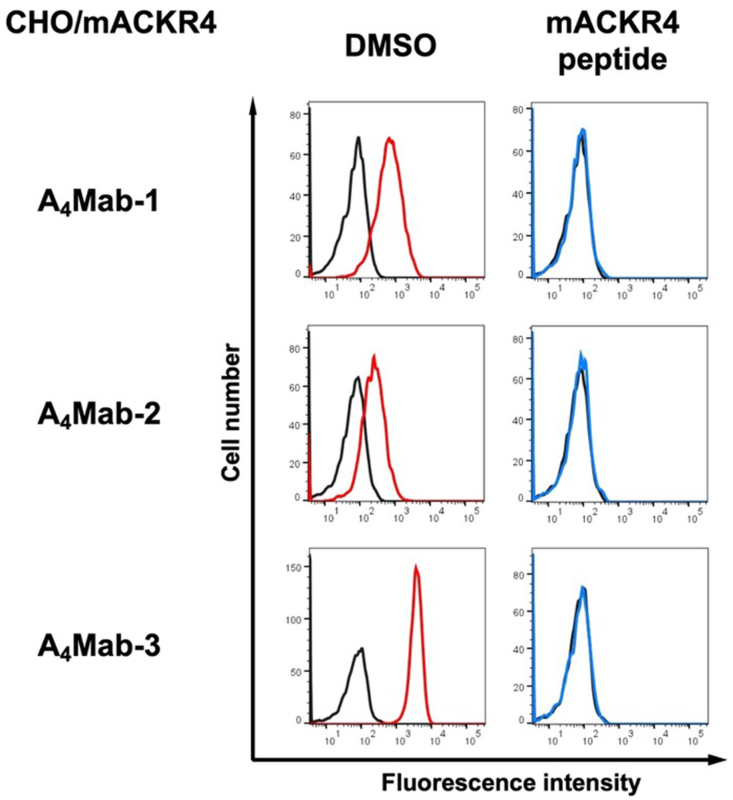
**A peptide-blocking assay using A**_**4**_**Mab-1, A**_**4**_**Mab-2, and A**_**4**_**Mab-3 with mACKR4 peptide.** A_4_Mab-1, A_4_Mab-2, or A_4_Mab-3 (0.1 μg/mL) plus mACKR4 peptide (10 μg/mL, blue line) or control (1 % DMSO in blocking buffer, red line) were reacted with CHO/mACKR4 for 30 min at 4 °C, followed by treatment with Alexa Fluor 488-conjugated anti-rat IgG. The black line represents the negative control (blocking buffer). DMSO, dimethyl sulfoxide.

We next performed a peptide-blocking assay. As shown in Fig. 2, A_4_Mab-1, A_4_Mab-2, and A_4_Mab-3 reacted with the CHO/mACKR4. The mACKR4 peptide wholly neutralized these reactions (Fig. 2).

### 3.3 Determination of the binding affinity of A_4_Mab-1, A_4_Mab-2, and A_4_Mab-3 using flow cytometry

**Fig. 3:**
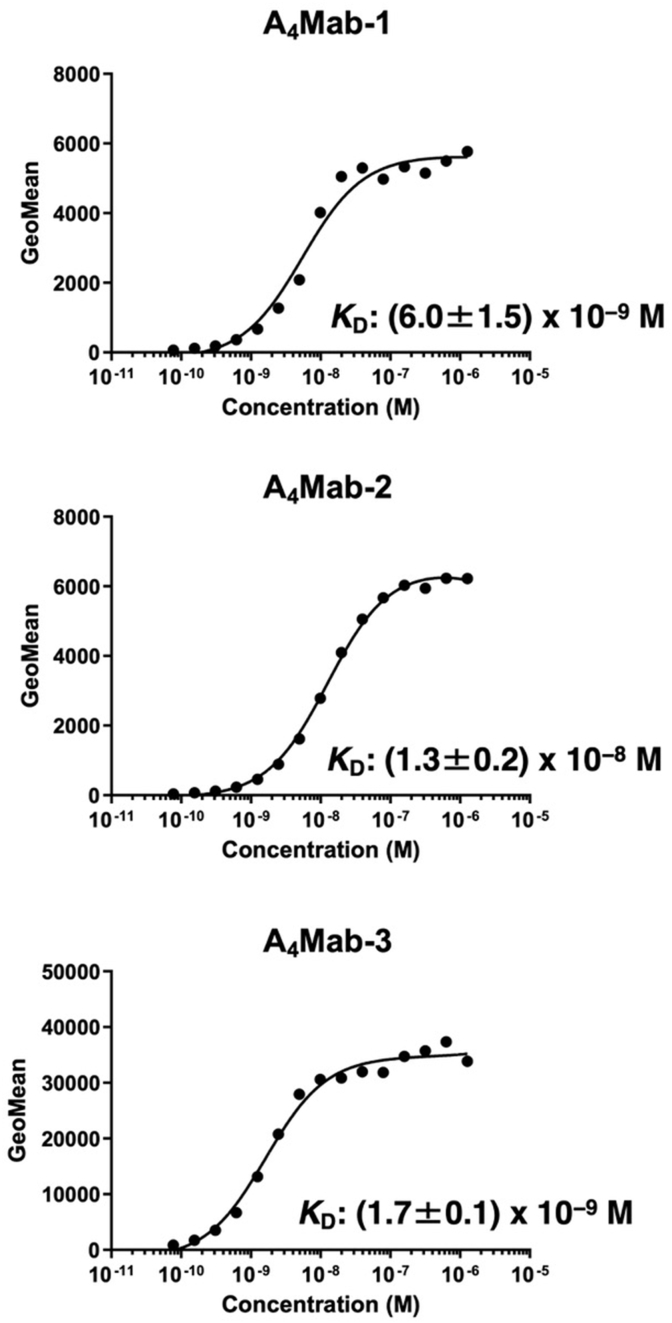
**The binding affinity of anti-mACKR4 mAbs.** CHO/mACKR4 cells were suspended in serially diluted A_4_Mab-1, A_4_Mab-2, or A_4_Mab-3. The cells were treated with anti-rat IgG conjugated with Alexa Fluor 488. The fluorescence data were subsequently collected using the SA3800 Cell Analyzer, followed by the calculation of the *K*_D_ using GraphPad PRISM 6. The experiments were performed three times independently and the representative results were shown. The *K*_D_ values (mean ± SD [M]) were determined from the three independent experiments.

To determine the *K*_D_ values of A_4_Mab-1, A_4_Mab-2, and A_4_Mab-3 against CHO/mACKR4, we conducted flow cytometry, and the geometric mean of the fluorescence intensity was plotted versus the concentration. The *K*_D_ values of A_4_Mab-1, A_4_Mab-2, and A_4_Mab-3 for CHO/mACKR4 were determined as 6.0 × 10^−9^ M, 1.3 × 10^−8^ M, and 1.7 × 10^−9^ M, respectively. (Fig. 3). These results indicate that A_4_Mab-3 possesses the most superior affinity to CHO/mACKR4.

### 3.4 Detection of mACKR4 using A_4_Mab-1, A_4_Mab-2, and A_4_Mab-3 by western blotting

**Fig. 4:**
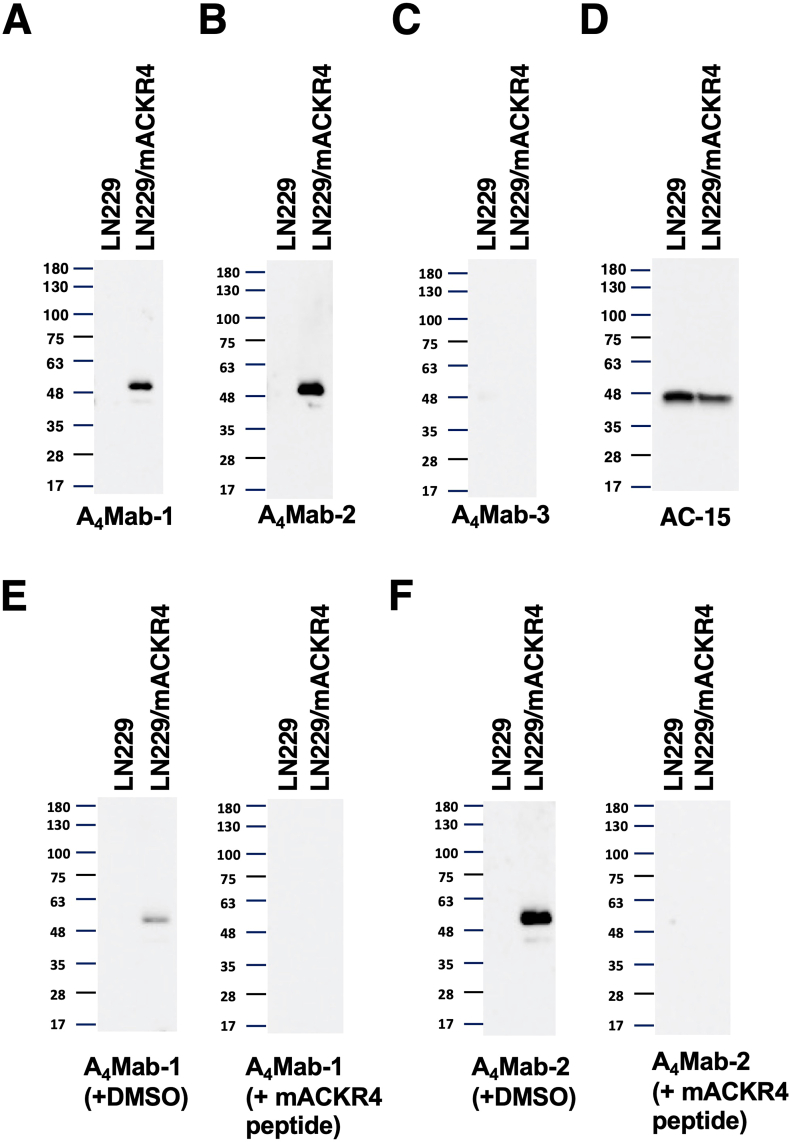
**Western blotting using anti-mACKR4 mAbs.** The lysates of LN229 and LN229/mACKR4 cells were electrophoresed and transferred onto polyvinylidene difluoride membranes. The membranes were incubated with 1 μg/mL of A_4_Mab-1 (A), 1 μg/mL of A_4_Mab-2 (B), 1 μg/mL of A_4_Mab-3 (C), or 1 μg/mL of AC-15 (an anti-β-actin mAb) (D). The membranes were subsequently incubated with peroxidase-conjugated anti-rat IgG (for A_4_Mab-1, A_4_Mab-2, and A_4_Mab-3) or anti-mouse IgG (for AC-15). Note that the exposure time of A_4_Mab-1, A_4_Mab-2, and A_4_Mab-3 blotting was the same. (E) The membranes were incubated with 1 μg/mL of A_4_Mab-1 plus the mACKR4 peptide (1 μg/mL) or DMSO. (F) The membranes were incubated with 1 μg/mL of A_4_Mab-2 plus the mACKR4 peptide (1 μg/mL) or DMSO. They were subsequently incubated with peroxidase-conjugated anti-rat IgG. These experiments were performed at least twice.

Western blotting was performed to assess the reactivity of A_4_Mab-1, A_4_Mab-2, and A_4_Mab-3. Lysates of LN229 and LN229/mACKR4 cells were probed. A_4_Mab-1 (Fig. 4A) and A_4_Mab-2 (Fig. 4B) detected mACKR4 as a ∼50-kDa band. In contrast, A_4_Mab-1 and A_4_Mab-2 did not show any bands from the lysates of LN229 cells. A_4_Mab-2 exhibited a superior reactivity against LN229/mACKR4 cell lysate compared to A_4_Mab-1 (Fig. 4A and B, the same exposure time). In contrast, A_4_Mab-3 could not detect any bands in the LN229/mACKR4 cell lysate (Fig. 4C). An anti-β-actin was used as an internal control (Fig. 4D). We also performed the peptide blocking experiment. We found that the 50-kDa band detected by A_4_Mab-1 and A_4_Mab-2 was blocked in the presence of mACKR4 peptide (Fig. 4E and F, respectively). These results suggest that A_4_Mab-1 and A_4_Mab-2 are useful for detecting mACKR4 by western blotting.

## 4 Discussion

In this study, we developed novel anti-mACKR4 mAbs (A_4_Mab-1, A_4_Mab-2, and A_4_Mab-3) using the N-terminal peptide immunization and showed the usefulness for flow cytometry (Fig. 1−3) and western blotting (Fig. 4) to detect mACKR4. Because no anti-mACKR4 mAb for flow cytometry has been reported, these mAbs could be the first ones for detecting mouse ACKR4 in flow cytometry. The information on anti-ACKR4 mAbs is available from the Antibody Bank of Tohoku University (http://www.med-tohoku-antibody.com/topics/001_paper_antibody_PDIS.htm#ACKR4).

ACKR4 has emerged as an essential regulator of DC migration via CCR7, as ACKR4 binds to the CCR7 ligands, CCL19 and CCL21 [31]. ACKR4 does not induce classical GPCR signaling and instead leads to the degradation of chemokine [32,33]. ACKRs are linked to endocytic machinery via β-arrestin. Upon chemokine ligation to ACKRs, the complex is generally internalized to endosomal machinery and subsequent chemokine degradation by lysosome [34]. A_4_Mab-1, A_4_Mab-2, and A_4_Mab-3 recognize the N-terminal region of mACKR4 with different *K*_D_ values from 1.3 × 10^−8^ M to 1.7 × 10^−9^ M (Fig. 3). Although the ACKR4 N-terminus has not been determined as a ligand-binding region, it would be interesting to investigate whether these mAbs possess the neutralizing activity to the ligands or promote the internalization of mACKR4. These functions are expected to inhibit the mACKR4 activity, which may increase the number of mACKR4 ligands.

A_4_Mab-1, A_4_Mab-2, and A_4_Mab-3 are applicable for flow cytometry (Fig. 1−3), and A_4_Mab-1, A_4_Mab-2, but not A_4_Mab-3 are useful for western blotting (Fig. 4). These results suggest that A_4_Mab-3 possesses the different epitope from A_4_Mab-1 and A_4_Mab-2. Previously, we determined the epitope of Cx_6_Mab-1 (an anti-mouse CXCR6 mAb) using 1 × and 2 × alanine scanning methods [35]. In future studies, we should evaluate the epitopes of A_4_Mab-1, A_4_Mab-2, and A_4_Mab-3. The identification of epitopes would contribute to understanding the properties of mAbs.

Immune checkpoint blockades have developed for cancer treatments [36]. The extent of intratumor CD8^+^ cell infiltration is correlated with improved responsiveness of anti-PD-1/PD-L1 therapy in melanoma [37] and improved outcomes in colorectal cancer [38]. A study investigated the roles of host mACKR4 in the development of mammary tumor by crossing MMTV-PyMT transgenic mice with the mACKR4-knockout strain [12]. The mammary tumor development was delayed in the mACKR4-knockout compared to control mACKR4-sufficient transgenic mice [12]. Mechanistically, the loss of mACKR4 leads to increased intra-tumor CCL21 levels and elevated numbers of CD103^+^ DCs and CD8^+^ cells within tumors. However, the contribution of mACKR4-expressing host cells to tumor microenvironment (TME) has not been investigated. Therefore, A_4_Mab-1, A_4_Mab-2, and A_4_Mab-3 would help identify the host cells that contribute to the formation of immunosuppressive TME. Furthermore, these mAbs could contribute to the preclinical studies for the depletion of mACKR4-expressing cells to enhance responsiveness to immune checkpoint blockade or T-cell co-stimulation.

## Author disclosure statement

The authors have no conflict of interest.

## Funding information

This research was supported in part by 10.13039/100009619Japan Agency for Medical Research and Development (AMED) under Grant Numbers: JP24am0521010 (to Y.K.), JP24ama121008 (to Y.K.), JP23am0401013 (to Y.K.), JP24bm1123027 (to Y.K.), and JP24ck0106730 (to Y.K.), and by the 10.13039/501100001691Japan Society for the Promotion of Science (JSPS) Grants-in-Aid for 10.13039/501100001691Scientific Research (KAKENHI) grant nos. 22K06995 (to H.S.), 21K20789 (to T.T.), 21K07168 (to M.K.K.), and 22K07224 (to Y.K.).

## CRediT authorship contribution statement

**Miu Hirose:** Investigation. **Hiroyuki Suzuki:** Writing – original draft, Investigation, Funding acquisition. **Rena Ubukata:** Investigation. **Tomohiro Tanaka:** Investigation, Funding acquisition. **Mika K. Kaneko:** Funding acquisition, Conceptualization. **Yukinari Kato:** Writing – review & editing, Project administration, Funding acquisition, Conceptualization.

## Declaration of competing interest

The authors declare the following financial interests/personal relationships which may be considered as potential competing interests: Yukinari Kato reports financial support was provided by 10.13039/100009619Japan Agency for Medical Research and Development. Hiroyuki Suzuki reports financial support was provided by 10.13039/501100001691Japan Society for the Promotion of Science. Mika K. Kaneko reports financial support was provided by 10.13039/501100001691Japan Society for the Promotion of Science. Tomohiro Tanaka reports financial support was provided by 10.13039/501100001691Japan Society for the Promotion of Science. If there are other authors, they declare that they have no known competing financial interests or personal relationships that could have appeared to influence the work reported in this paper.

## Figures and Tables

**Figure:**
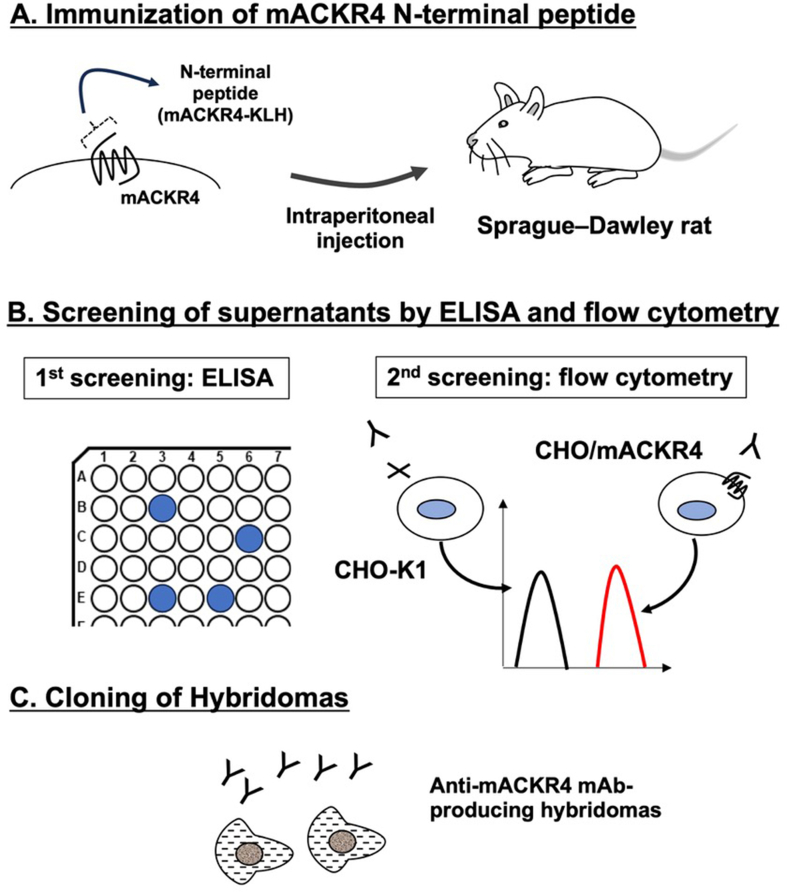


**Figure:**